# End-of-life care for people with severe and persistent mental illness and a life-limiting disease: An umbrella review

**DOI:** 10.1192/j.eurpsy.2025.2440

**Published:** 2025-03-24

**Authors:** Jonas Denduyver, Johan Detraux, Justien Weydts, Marc De Hert

**Affiliations:** 1 University Psychiatric Center Katholieke Universiteit Leuven, Kortenberg, Belgium; 2 Department of Neurosciences, Center for Clinical Psychiatry, Katholieke Universiteit Leuven, Leuven, Belgium; 3 Leuven Brain Institute, Katholieke Universiteit Leuven, Leuven, Belgium; 4 Antwerp Health Law and Ethics Chair, University of Antwerp, Antwerp, Belgium

**Keywords:** advance care planning, bipolar disorder, end-of-life care, integration of care, schizophrenia, severe and persistent mental illness, stigma

## Abstract

**Background:**

It is widely known that people with a severe and persistent mental illness (SPMI) are more at risk of poor physical health outcomes because of disparities in healthcare access and provision. Less is known about the quality of end-of-life (EoL) care in people with SPMI who have a life-limiting disease.

**Methods:**

A comprehensive and systematic literature search in PubMed, Embase, Web of Science, Scopus, and CINAHL electronic databases (from inception to November 2023) was conducted, without language restriction, for reviews on EoL care and/or palliative sedation for people with SPMI and a life-limiting disease. A critical appraisal of the selected reviews was performed. Data were analyzed according to the four principles of biomedical ethics.

**Results:**

Ten reviews were included. These show that people with SPMI are at risk of suboptimal EoL care. Stigma among healthcare professionals, lack of integrated care policies, absence of advanced care planning, and insufficient expertise and training in palliative care of psychiatrists have been identified as key challenges to the provision of adequate EoL care for people with SPMI. No data were found about palliative sedation.

**Conclusions:**

To optimize palliative and EoL care for SPMI patients with a life-limiting disease, a policy of coordinated and integrated mental and physical healthcare is needed. Moreover, education and training initiatives to reduce stigma and discrimination among all healthcare workers and to enhance palliative care skills in psychiatrists should be offered. Finally, more research is needed on EoL particularly on palliative sedation for people with SPMI and a life-limiting disease.

## Introduction

Severe and persistent mental illness (SPMI) is a term used to define persons who experience serious and enduring functional impairment as a result of a mental, behavioral, or emotional disorder [1–3]. Although the definition of SPMI is contested [1, 2], it mostly includes schizophrenia, bipolar disorder, and recurrent or major depressive disorder [1, 4]. According to a 2018 report by the Organisation for Economic Co-operation and Development (OECD), in Europe, approximately 27.5 million individuals were affected by at least one of these diagnoses [5].

Individuals with SPMI have a higher risk of somatic comorbidities, such as cardiovascular disease, respiratory disease, or cancer [6–10]. Contributing factors include unhealthy lifestyle, long-term intake of psychotropic drugs, and disparities in healthcare access and provision [7, 11–14]. As a result, these persons tend to die 10 to 20 years earlier than the general population [15–17]. When suffering from a life-limiting disease, SPMI patients are, like others, entitled to qualitative palliative care, most particularly at the end of life (EoL) [18, 19]. Palliative and EoL care have the purpose to improve the quality of life of the patient, their family, and caregivers despite their suffering from a severe and progressing illness [18]. This is achieved through managing physical symptoms and providing psychosocial and spiritual support [18, 20]. Palliative sedation is an integral part of palliative care and is used to resolve or alleviate refractory symptoms (e.g., intractable pain) at the EoL [21].

Although there is sufficient evidence that disparities in healthcare access and provision contribute to poor physical health outcomes in people with SPMI, less is known about the quality of EoL care in these individuals [22]. The existing systematic reviews on this topic are very disparate, using different methodologies, and discussing divergent issues. The goal of this umbrella review therefore is to identify and analyze the most pertinent issues in this domain from a biomedical ethics perspective. Furthermore, a quality appraisal of the included reviews was performed.

## Methods

### Search strategy

A comprehensive and systematic literature search in PubMed, Embase, Web of Science, Scopus, and CINAHL electronic databases (from inception to November 2023), without language restriction, was conducted for systematic reviews (scoping reviews, mapping reviews, meta-analyses, integrative reviews, and umbrella reviews) examining the organization of palliative/hospice/EoL care for people with an SPMI. JD, closely working together with another experienced biomedical information specialist, constructed search strings for the different databases. The preliminary keywords that were used to perform these searches included the following: (schizophrenia OR depressive disorder OR unipolar disorder) AND (palliative care OR end-of-life care) AND (systematic review OR scoping review OR meta-analytic review). A self-developed filter was added to the search strings for retrieving all kinds of reviews using a systematic search strategy [23]. Full search strategies are available as Supplementary Material. Duplicates were removed by JD, using Endnote X9 and Rayyan. After removing duplicates, titles and abstracts were screened independently by JW and JDD, using Rayyan. JDD and MDH did the full-text screening independently. Articles that were deemed potentially relevant according to the selection criteria were included. Any doubts were solved by consensus. References of the selected studies and pertinent reviews were carefully cross-checked for other relevant studies.

### Eligibility criteria

#### Inclusion criteria

Any review systematically researching literature on EoL care and/or palliative sedation for people with SPMI (i.e., people with a schizophrenia spectrum and other psychotic disorders, bipolar and related disorders, or a recurrent or currently severe major depressive disorder) in the context of a life-limiting physical illness was included.

#### Exclusion criteria

Reviews on “palliative psychiatry” (medical futility, euthanasia, or physician-assisted suicide cases), mental health problems as a consequence of EoL, neurodegenerative diseases (such as dementia), intellectual disability or substance abuse (except where these coexisted with SPMI), or homeless people without SPMI, as well as viewpoints, recommendations, editorials, reviews that were not peer-reviewed or published (preprints, dissertations, conference abstracts/papers, books/book sections, grey literature), or study protocols were excluded.

### Quality appraisal

The included studies were appraised by JDD, using the Joanna Briggs Institute (JBI) Critical Appraisal tool for Systematic Reviews [24].

### Data extraction

Data were extracted and mapped descriptively by JDD by using the JBI Data Extraction Form for Review for Systematic Reviews and Research Syntheses [24]. This extraction form included the following information: author(s) and year of publication; countries where the included studies were conducted as well as the range (years) of the included studies; studied topic(s) of interest; nature of literature (empirical, peer-reviewed, and grey literature), patient characteristics (age, clinical diagnosis); outcomes/key findings that relate to the review questions. Full data extraction is available as Supplementary Material.

## Results

### Search strategy

**Figure 1.**
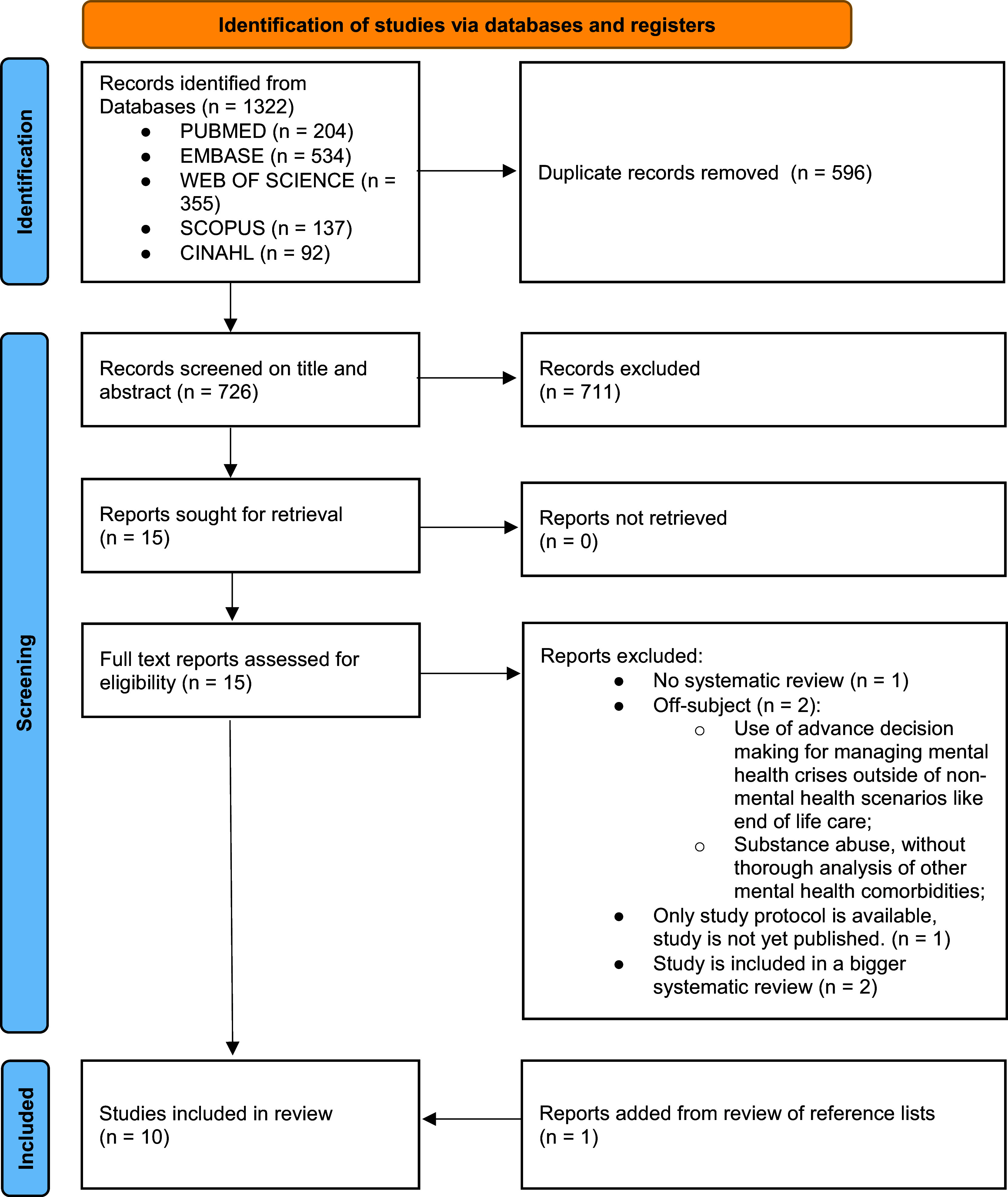
Primsma flow chart.

The search in PubMed (n = 204), Embase (n = 534), Web of Science (n = 355), Scopus (n = 137), and CINAHL (n = 92) yielded a total of 1,322 reports. Of these, 596 duplicate reports were removed. Overall, 726 records were selected as potentially eligible, of which 10 original records met the inclusion criteria. The results of the study selection are shown in the PRISMA flow diagram (see Figure 1) [25].

### Study and patient characteristics

The search yielded 10 systematic reviews. Three identified themselves as “scoping” reviews [26–28], though all reviews applied a scoping review in the sense that they all developed a systematic search strategy and mapped the body of literature on a certain topic area within this field. The scoping review question(s) of course differs across the included scoping reviews. Five reviews included “grey literature” [4, 26–29].

All articles were published in English. Three studies were conducted in both Canada [4, 27, 28] and the USA [30–32], 2 in the UK [29, 33], 1 in Australia [26] and 1 in the Netherlands [34]. Except for 1 [4], all articles were published in 2019 or later.

Eight reviews focused on people with an SPMI [4, 26, 27, 29–31, 33, 34]. While some did not further specify this term, others mentioned specific diagnoses. Two reviews only included people with schizophrenia [28, 32].

One review searched for any specific tools or interventions to improve palliative care for people with SPMI [34]; 1 partly focused on the organization of the Australian “National Disability Insurance System” (NDIS) [26]; and 1 limited their search to the place of death and healthcare utilization in the last year of life by people with SPMI [33]. None of the reviews reported on “palliative sedation.”

An overview of the study characteristics is presented in Table 1.

**Table 1.** Study characteristics

References	Country	Phenomenon of interest	Type of literature included	Number of literature	Chronological range of literature	Country of included literature	Patient age	Patient diagnosis
Boschen et al. [26]	Australia	Death, dying, and palliative care experiences (with special attention to Australian NDIS participants)	“Academic and grey literature”	66	2013–2021	Not specified	>18 years old	SPMI, bipolar disorder, schizophrenia, schizo-affective disorder, and psychosocial disability.
Hannigan et al. [29].	UK	End-of-life care	Empirical literature, case report, and grey literature	104	1983–2019	USA (40), Canada (7), UK (7), Australia (5), the Netherlands (3), France (2), Belgium (1), Ireland (1), Israel(1), Mexico (1), New Zealand (1), Singapore (1), Taiwan (1), and Unknown (1). Policy and guidance material was UK-only	>18 years old	SMI. Including but not limited to schizophrenia, schizophrenia spectrum and other psychotic disorders, schizotypal and delusional disorders, bipolar affective disorder, bipolar and related disorders, major depressive disorder, and disorders of adult personality and behavior.
Riley et al. [31]	USA	End-of-life care	Empirical literature only	9	2005–2019	USA (3), Australia (1), Canada (1), New Zealand (1), the Netherlands (1), Switzerland (1), and UK (1)	No age limit	SPMI, not further specified.
Baruth et al. [32]	USA	End-of-Life care	Empirical literature, review, and case report	33	2008–2018	USA (13), Canada (4), Australia (2), Belgium (2), Japan (2), Taiwan (2), UK (2), Austria (1), France (1), the Netherlands (1), Poland (1), and Sweden (1)	No age limit	Schizophrenia.
Hanan et al. [30]	USA	End-of-life care	Empirical literature only	8	2003–2018	USA (4), Canada (2), New Zealand (1), and Taiwan (1)	No age limit	SPMI or specifically schizophrenia.
Wilson et al. [33]	UK	Healthcare utilization in the last year of life/Place of death	Empirical literature only	23	1977–2019	USA (8), Canada (3), Australia (2), Denmark (2), Taiwan (2), UK (2), France (1), Japan (1), the Netherlands (1), and New Zealand (1)	>18 years old	SMI, not further specified.
Den Boer et al. [34]	Netherlands	Tools or interventions for optimalization of end-of-life care	Empirical literature only	4	2005–2016	USA (3) and the Netherlands (1)	No age limit	SPMI. Focus on psychotic disorders, bipolar disorder, and mood disorders.
Donald et al. [27]	Canada	End-of-Life Care	Empirical literature, review, case report, and theoretical analysis	46	2000–2018	USA (20), Australia (7), Canada (6), Europe (5), UK (5), New Zealand (1), South Africa (1), and Taiwan (1)	>18 years old	SPMI. Including schizophrenia, bipolar disorder, and major depression.
Relyea et al. [28]	Canada	End-of-life care	Empirical literature, review, case report, editorial, and grey literature	32	1983–2017	Not Specified	No age limit	Schizophrenia or schizo-affective disorder.
Woods et al. [4]	Canada	End-of-life care	Empirical literature, narrative review, case report, expert opinion, editorial, and commentary	68	1979–2007	USA (38), UK (10), Canada (9), Australia (2), and others (9)	No age limit	Mental disorders. Focus on schizophrenia, bipolar disorder, and severe depression. Comments on personality disorder, PTSD, anorexia nervosa, and alcoholism were also included.

### Quality appraisal

Overall, 2 reviews had a negative score on at least 4 of the 11 criteria. One review did not use an appropriate search strategy [30]. Three reviews did not use a data extraction tool [30–32]; for 1 review it was unclear which methods were used to minimize errors in data extraction [4]. In general, the methods used to combine studies were appropriate. However, not all reviews made appropriate recommendations for future practice, policy, or research: they were too brief, were not supported by the reported data, or were completely absent.

The reviews of Hannigan (MENLOC) and den Boer were the only two meeting all quality criteria [29, 34]. Accordingly, the MENLOC review is the most cited in our results.

Results of the quality appraisal are available as Supplementary Material.

### Main results

We used the framework provided by Beauchamp and Childress in their book on the four principles of biomedical ethics to describe the data from the included reviews [35]. The principle of justice constitutes the right of every patient to be treated equally, in daily practice and research. The principle of autonomy represents the right of the patient to make an informed decision about their own treatment. The principle of non-maleficence not only implies that clinicians need to refrain from actively harming the patient, but also need to avoid harm through neglect or ignorance. Finally, clinicians must actively act in the best interests of the individual patient (principle of beneficence). Some data, however, may be classified under multiple categories as there is an obvious overlap between these principles.

### Principle of justice

#### Allowing equal access

Several reviews indicate that individuals with SPMI do not have equal access to appropriate EoL care, compared with the general population [26, 27, 29–33]. For example, in the last month of life patients with SPMI receive less chemotherapy and fewer diagnostic tests [33]. Moreover, according to other reviews, some of these patients are more at risk of futile medical interventions, as they are admitted to intensive care and receive invasive treatment (cardiopulmonary resuscitation, mechanical ventilation, and parenteral nutrition) more often during the last months of their life, than people from the general population [26, 30, 32, 33].

This inequality in healthcare is strongly affected by stigmatization and discrimination [26–28]. Furthermore, Being confronted with negative experiences in healthcare, such as discrimination, reinforces people with SPMI in their healthcare-avoiding behavior [26, 27].

Some reviews indicate that another possible cause of this inequality is the way in which healthcare is organized. In most cases, there is no integration of mental health and palliative care [29, 30]. Palliative care is usually linked to acute somatic care in general hospitals [29], and is often focused on clearly defined populations, explaining why this service is limited for individuals with co-occurring mental disorders and serious physical problems [26, 27, 30, 31]. In the absence of a policy of coordinated and integrated mental and somatic healthcare, these people will fall through the cracks. This phenomenon is referred to as “system siloing” [4, 26–31].

#### Representing the vulnerable

Multiple reviews mention the lack of representation of the patient with SPMI, both at the policy and the individual level. The MENLOC study, a mixed methods systematic review, states that there is little attention to agents to help the patient in making healthcare decisions during EoL. Although the law states that everyone is entitled to a “healthcare proxy,” they found, for example, that only 1 out of 334 patients with SPMI actually had a proxy [29]. People with SPMI have a smaller personal network that can represent their interests, making them even more vulnerable [4, 26–31]. Although professionals can be called upon to assist a person with SPMI, these are not always well aware of the personal wishes and specific needs of the patient, and are not always committed [4, 26–28, 30]. This increases the risk of ill-informed decisions or decisions disregarding the personal preferences of the patient [4, 26, 29, 30].

#### Doing research

When it comes to EoL care, there is a clear research gap regarding people with SPMI [26]. One systematic review showed that research conducted at the interface between cancer and mental health primarily focuses on the mental impact of cancer on individuals without pre-existing SPMI [32]. Another review showed that existing research on EoL care in people with SPMI is very disparate, making it difficult to draw major conclusions [31]. Finally, very little research has been done on the patient’s perspective [4, 26].

Several reviews showed that few studies have been conducted on the development of appropriate clinical or policy tools in the context of EoL care for people with SPMI [4, 26–28, 31, 34]. Two of these discuss the feasibility of the Health Care Preferences Questionnaire (HCPQ), a tool to identify the needs and preferences of the patient concerning EoL care and making an advance care planning (ACP). However, figures on the impact of using these instruments on the quality of EoL care are still lacking [34].

### Principle of respect for autonomy

#### Starting the conversation

Reviews indicate that professionals tend to avoid talking about the approaching EoL with SPMI patients [4, 26, 28, 29]. They fear these conversations could be emotionally destabilizing and induce suicidal thoughts [4, 26, 29]. In addition, it is often thought that the patient will not entirely comprehend what is said. People with SPMI, however, do not experience these conversations as more disturbing and share the same concerns as others [4, 27, 31, 32, 34].

#### Respecting another point of view

Depending on their health state, patients with SPMI possibly can react in an unpredictable or unexpected way to EoL conversations [4, 28, 32]. They can act overly dependent, very dismissive, or even aggressive toward the caregiver [4, 26–32].

However, not every patient who refuses contact or further treatment can be classified as “inadequate.” The refusal can indeed be well-considered and well-founded [4, 26, 28, 29, 32]. Some reviews recommend that in cases where there is a genuine lack of clarity about the patient’s decision-making capacity, it may be appropriate to consult an ethics committee [29, 32]. Nevertheless, the MENLOC study mentions the importance of healthcare professionals (HCPs) to at all times be aware of their own emotions when evaluating patients’ decisions about the EoL process, as this can affect clinical and therapeutic functioning [29].

#### Giving the patient control

Several reviews point to the fact that it must always be assumed that a person has decision-making capacity until proven otherwise. Moreover, every effort must be made to promote decision-making capacity [28, 29, 34].

In daily practice, HCPs sometimes assume patients are incompetent to make decisions solely on the basis of them having a psychiatric illness. As a result, people with SPMI are less likely to be involved in conversations about making medical decisions or ACP than the average population. In these cases, HCPs address relatives or substitute decision-makers directly [4, 26–30]. Some reviews state, however, that it is important to realize that decision-making capacity is always linked to specific decisions and situations, not to medical diagnoses [4, 32]. Data from reviews also demonstrate that patients with SPMI want to be involved in decisions about EoL [4, 26, 28–30], but rarely start the conversation about this subject [29, 30].

#### Appreciating the importance of a homely environment

Reviews clearly indicate that people with SPMI, like the general population, want to spend the end of their lives at home or in a familiar environment [26, 29]. EoL care can take place in any setting that the person with SPMI considers their home: a sheltered housing initiative, nursing home, and homeless shelter.[28]

Several reviews report data of studies concerning the place of death and the use of healthcare by individuals with SPMI during the last months of their life. It remains unclear whether the number of deaths in hospital, in comparison to the number of deaths at home, is greater in the SPMI population than the general population [29, 33]. Despite this lack of data, it seems that people with SPMI die in a nursing home more often than the general population [28–33].

Reviews also point to the fact that individuals with SPMI, who have been in a mental health facility for a large part of their lives, cannot stay in this facility when care for the life-limiting disease becomes too complex. In these circumstances, they are referred to somatic services, where specialized care can be provided [26, 29]. Not only are these people displaced from their familiar environment but also they have to part from the care staff who in many cases are their main confidants [26–28, 32]. In the most unfortunate cases, patients are referred back and forth between mental and somatic care settings, because of the complex care needs [4, 26–29, 31].

### Principle of non-maleficence

#### Referring to specialized care

Reviews indicate that HCPs experience difficulties in referring a patient with SPMI to specialized EoL care in a timely and adequate manner. Besides stigma and prejudice [4, 26, 28–30], lack of psychiatric knowledge and feeling for psychiatric patients, and challenging communication and data transfer problems (absence of information on the psychiatric history of the patient) further complicate adequate EoL care after referral [26, 27, 29, 31].

#### Teaching your colleagues

The majority of mental health professionals have no experience with EoL care. As a consequence, they are insecure about providing EoL care and tend to avoid it. They are afraid of doing things that fall outside the legal framework because they lack the required knowledge [4, 26, 28, 29, 31] and institutional guidelines regarding the provision of palliative care in mental health settings [26, 27, 31, 32].

Palliative care providers, on the other hand, are uncertain when it comes to supporting people with SPMI. They feel uncomfortable and find they lack the knowledge, training, skills, and experience to cope with these patients [4, 26, 28, 29, 31]. They also experience difficulties in dealing with behavioral problems [27].

Various reviews argue that there is great benefit in cross-training between HCPs [4, 27–29]. Appointing a psychiatric liaison worker to a palliative care unit, or vice versa, can help to increase the expertise and self-confidence of HCPs, through support, education, and supervision [4, 26, 27, 29, 31]. Specific training for palliative care providers can focus on knowledge of the most common psychiatric syndromes, skills for a global psychiatric assessment, debunking prejudice, and dealing with difficult behavior [4, 28, 29]. Training for mental health professionals can focus on making an ACP and on grief counseling for bereaved relatives [28, 29]. The MENLOC study suggests it is preferable that the liaison worker has direct contact with the patient and is an integral part of daily clinical practice [29].

#### Working in team

Besides cross-training between services, several reviews suggest that an intrinsic understanding between mental health and palliative care facilities – both intramural and extramural – and the integration of team members from both healthcare services also benefit the patient with SPMI [4, 28, 29]. One suggestion is a regular multidisciplinary meeting to discuss specific cases [27–29, 32]. An even bigger step is an integrated unit involving all relevant specializations (e.g., psychiatrist, oncologist) [4, 26, 27, 29, 32].

### Principle of beneficence

#### Acting with medical expertise

Several reviews point out that the complex pathology of individuals with SPMI and serious somatic comorbidities make heavy demands on the available time of HCPs. Broad medical expertise is recommended [27, 32]. However, the growing fragmentation of medical knowledge into ever more specialized sub-disciplines adds to the difficulty in the provision of adequate care.

There is a great risk that physical deterioration is not noticed in time because some SPMI patients have a disturbed body perception (reduced pain sensitivity) and communication deficits [4, 26–30, 32], or because physicians misattribute physical symptoms to the mental illness [4, 26, 28, 32].

Several reviews indicate that special attention should be paid by the clinician to the adequate management of medication. The interaction between palliative medication (pain control, chemotherapy) and psychotropic drugs (antipsychotics, antidepressants) can potentially cause serious side effects [27–29].

#### Supporting relatives

Some reviews emphasize the importance of supporting the patient’s relatives [27]. Families including a person with SPMI are often characterized by more family conflicts, a complex family structure, and family members who are struggling with mental illnesses themselves [29, 31]. Sometimes, relatives are designated as substitute decision-makers and are in need of support by HCPs when they are in the position to effectively make important decisions on behalf of the patient [26, 28, 29, 32].

Family members, as well as the patient, want to stay in touch with the same team that has already been caring for the patient for years, and not having to discuss EoL problems or issues with other care providers. In this way, concerns are more easily discussed and solved [28].

One review mentions the importance of providing appropriate support groups for bereaved relatives, following the death of the patient with SPMI [28].

## Discussion

We conducted this umbrella review to identify and analyze the most pertinent issues concerning EoL care for people with SPMI and a life-limiting disease from a biomedical ethics perspective. This synthesis revealed that several issues complicate the application of ethical principles in EoL care for this population. Key challenges identified include stigma among HCPs, a lack of integrated care policies, the absence of ACP, and insufficient expertise and training of psychiatrists in palliative care.

These issues are not unique to EoL care but reflect broader systemic challenges [36]. Disparities in general healthcare access, quality, and outcomes for people with SPMI are well-documented and widespread [7, 13]. In high-income countries with specialized healthcare systems, people with complex pathologies often fall through the cracks [37], particularly when these complex conditions lead to more atypical behavior and social decline, as seen in people with SPMI [38, 39].

In a general healthcare setting, the issue of stigma surrounding severe mental illness contributes to diagnostic overshadowing, delayed diagnosis, and less direct contact between patients and HCPs [14, 40–42]. Additionally, the lack of coordination between mental and somatic healthcare results in delayed, fragmented care, and in worse health outcomes [38, 43, 44]. Even within the mental healthcare system, the needs of people with SPMI are insufficiently met due to the issues mentioned above [45, 46]. As a result, patients with SPMI experience high numbers of readmissions, emergency visits, and coercive interventions [47].

These challenges underscore the urgent need for a shift in how healthcare systems approach the care of individuals with SPMI, particularly in the context of EoL care. Current healthcare models often fail to address the complex and nuanced needs of this population [48]. The integration of models such as community-based mental healthcare and palliative psychiatry could help address these challenges.

Community-based mental healthcare models, such as assertive community treatment (ACT), prioritize psychosocial rehabilitation over symptom management alone [49]. In these models, patients receive follow-up from outreaching healthcare teams, who can help bridge the gap between services and challenge implicit stigmatizing attitudes and behaviors toward SPMI among other HCPs [50–52].

Palliative psychiatry, an emerging field grounded in the values of palliative care, specifically addresses the needs of the SPMI population, who often suffer from symptoms unresponsive to standard treatments [18, 53]. Palliative principles, such as reducing harm, alleviating suffering, respecting the autonomy of the patient, and maintaining dignity, are applied regardless of whether a life-limiting illness or death is imminent [54]. One way to achieve these principles is to make a comprehensive ACP at an early stage [55, 56]. This psychiatric advance directive (PAD) addresses the psychological needs of the patient during times when their decision-making capacity fluctuates due to their psychiatric condition, but also integrates EoL preferences such as wishes regarding life-saving interventions or invasive procedures [57–61].

By adopting care models suited to the needs of individuals with SPMI, many of the ethical challenges faced in palliative care could be addressed, ultimately improving both the quality and dignity of care provided to this vulnerable population.

This umbrella review has *strengths and limitations.* A key strength of this analysis certainly is the extensive search strategies including several databases (see Supplementary Material). An important limitation is that all reviews included in this umbrella review are based on studies that have been conducted in high-income countries with developed health systems. Moreover, none of the reviews addressed the use of palliative sedation among SPMI patients. To address this limitation we conducted an additional search for individual empirical studies on this subject but found no relevant articles. Finally, although umbrella reviews certainly represent one of the highest levels of evidence synthesis currently available, a major limitation is that these reviews only report what researchers have systematically reviewed [62]. Therefore, more recent articles or empirical studies might have been missed.

## Conclusion

Care for individuals with SPMI poses a multifaceted and complex challenge. Particularly in the context of EoL care when confronted with a life-limiting disease. There remains an urgent need for a policy of coordinated and integrated mental and physical healthcare for people with SPMI and a life-limiting disease. Moreover, education and training initiatives to address blind spots of psychiatric as well as palliative care providers need to be developed. Finally, we specifically draw attention to the fact that no scientific data on the use of palliative sedation in the SPMI population were found. Therefore, more research is needed, especially in relation to the practice of ACP.

## Supporting information

Denduyver et al. supplementary material 1 Denduyver et al. supplementary material

Denduyver et al. supplementary material 2 Denduyver et al. supplementary material

Denduyver et al. supplementary material 3 Denduyver et al. supplementary material

## Data Availability

The analysis is based on the content of the selected publications. Data extraction and coding can be found in Supplement 3.

## Supplementary material

The supplementary material for this article can be found at http://doi.org/10.1192/j.eurpsy.2025.2440.
