# Supplementary material for: End-of-life care for people with severe and persistent mental illness and a life-limiting disease: An umbrella review
Source: Eur Psychiatry. 2025 Mar 24;68(1):e49. doi: 10.1192/j.eurpsy.2025.2440 (PMC12041735; doi:10.1192/j.eurpsy.2025.2440)
Supplement: Denduyver et al. supplementary material 1 — Denduyver et al. supplementary material [file S092493382502440Xsup001.docx]

**PUBMED (3209 hits, 26/12/2022, 178 met filter)**

**PUBMED (3392 hits, 06/11/2023, beginning - 06/11/2023, 204 hits met eigen filter, update 01/12/2022 - 06/11/2023, 29 hits met eigen filter)**

("Schizophrenia Spectrum and Other Psychotic Disorders"[Mesh] OR "schizophreni*"[tiab] OR "schizoaffective*"[tiab] OR "schizo-affective*"[tiab] OR "delusional disorder*"[tiab] OR "paranoid disorder*"[tiab] OR "paranoid psychosis"[tiab] OR "schizotypal disorder*"[tiab] OR "psychotic disorder*"[tiab] OR "psychosis"[tiab] OR "catatoni*"[tiab] OR "Bipolar and Related Disorders"[Mesh] OR "Mood Disorders"[Mesh] OR "bipolar*"[tiab] OR "cyclothymi*"[tiab] OR "depressive disorder*"[tiab] OR "depressive disease*"[tiab] OR "depressive illness*"[tiab] OR "depressive episode*"[tiab] OR "depressive state*"[tiab] OR "depressive syndrome*"[tiab] OR "depression*"[tiab] OR "unipolar disorder*"[tiab] OR "manic episode*"[tiab] OR "mania"[tiab] OR "affective disorder*"[tiab] OR "mood disorder*"[tiab] OR "MDD"[tiab] OR "disruptive mood dysregulation disorder*"[tiab] OR "dysthymi*"[tiab] OR "disthymi*"[tiab] OR "dysthimi*"[tiab] OR "premenstrual dysphoric disorder*"[tiab] OR "severe mental illness*"[tiab] OR "serious mental illness*"[tiab] OR "severe mental disorder*"[tiab] OR "serious mental disorder*"[tiab]) AND ("Palliative Care"[Mesh] OR "palliative care*"[tiab] OR "palliative supportive care*"[tiab] OR "palliative treatment*"[tiab] OR "palliative therap*"[tiab] OR "Hospices"[Mesh] OR "hospice*"[tiab] OR "Hospice and Palliative Care Nursing"[Mesh] OR "palliative nursing"[tiab] OR "Palliative Medicine"[Mesh] OR "palliative medicine*"[tiab] OR "Terminal Care"[Mesh:NoExp] OR "terminal care*"[tiab] OR "end-of-life care*"[tiab] OR "EOL care*"[tiab] OR "Hospice Care"[Mesh]) AND (("systematic*"[tiab] AND "review*"[tiab]) OR ("systematic*"[tiab] AND "overview*"[tiab]) OR ("systemic*"[tiab] AND "review*"[tiab]) OR ("systemic*"[tiab] AND "overview*"[tiab]) OR "systematic literature review*"[tiab] OR "Cochrane review*"[tiab] OR "scoping review*"[tiab] OR "scoping overview*"[tiab] OR "scoping literature review*"[tiab] OR "mapping review*"[tiab] OR "systematic mapping*"[tiab] OR "systematical mapping*"[tiab] OR "umbrella review*"[tiab] OR "umbrella systematic review*"[tiab] OR "systematic umbrella review*"[tiab] OR "umbrella analys*"[tiab] OR "umbrella of systematic*"[tiab] OR "review of review*"[tiab] OR "overview of reviews"[tiab] OR "overview of systematic reviews"[tiab] OR "overviews of reviews"[tiab] OR "reviews in overviews"[tiab] OR "meta-review*"[tiab] OR "integrative review*"[tiab] OR "integrative systematic review*"[tiab] OR "integrated review*"[tiab] OR "integrated systematic review*"[tiab] OR "integrative overview*"[tiab] OR "meta-synthesis"[tiab] OR "metasynthesis"[tiab] OR "research synthesis"[tiab] OR "meta-ethnography"[tiab] OR "systematic literature search"[tiab] OR "systematic literature research"[tiab] OR "metaanalys*"[tiab] OR "meta-analys*"[tiab] OR "meta-analytic review*"[tiab] OR "meta-analytical review*"[tiab] OR "meta-analys*"[pt] OR "systematic review"[pt]) NOT ("letter"[pt] OR "editorial"[pt] OR "comment"[pt] OR "case reports"[pt] OR "historical article"[pt] OR "protocol*"[tiab] OR "withdrawn"[tiab] OR "retraction of publication"[pt] OR "retraction of publication as topic"[Mesh] OR "retracted publication"[pt] OR "reply"[tiab] OR "published erratum"[pt])

**EMBASE (9222 hits, 26/12/2022, 468 met eigen filter)**

**EMBASE (9870 hits, 06/11/2023, beginning - 06/11/2023, 534 hits met eigen filter, update 01/12/2022 - 06/11/2023, 79 hits met eigen filter)**

('psychosis'/de OR 'acute psychosis'/exp OR 'affective psychosis'/exp OR 'depressive psychosis'/exp OR 'endogenous psychosis'/exp OR 'manic psychosis'/exp OR 'paranoid psychosis'/exp OR 'schizophrenia spectrum disorder'/exp OR 'mood disorder'/exp OR 'disruptive mood dysregulation disorder'/exp OR 'psychosis':ti,ab,kw OR 'schizophreni*':ti,ab,kw OR 'schizoaffective*':ti,ab,kw OR 'schizo-affective*':ti,ab,kw OR 'delusional disorder*':ti,ab,kw OR 'paranoid disorder*':ti,ab,kw OR 'schizotypal disorder*':ti,ab,kw OR 'psychotic disorder*':ti,ab,kw OR 'catatoni*':ti,ab,kw OR 'mania':ti,ab,kw OR 'bipolar*':ti,ab,kw OR 'cyclothymi*':ti,ab,kw OR 'depressive disorder*':ti,ab,kw OR 'depressive disease*':ti,ab,kw OR 'depressive illness*':ti,ab,kw OR 'depressive episode*':ti,ab,kw OR 'depressive state*':ti,ab,kw OR 'depressive syndrome*':ti,ab,kw OR 'depression*':ti,ab,kw OR 'unipolar disorder*':ti,ab,kw OR 'manic episode*':ti,ab,kw OR 'affective disorder*':ti,ab,kw OR 'mood disorder*':ti,ab,kw OR 'MDD':ti,ab,kw OR 'disruptive mood dysregulation disorder*':ti,ab,kw OR 'dysthymi*':ti,ab,kw OR 'disthymi*':ti,ab,kw OR 'dysthimi*':ti,ab,kw OR 'premenstrual dysphoric disorder*':ti,ab,kw OR 'severe mental illness*':ti,ab,kw OR 'serious mental illness*':ti,ab,kw OR 'severe mental disorder*':ti,ab,kw OR 'serious mental disorder*':ti,ab,kw) AND ('palliative therapy'/exp OR 'palliative therap*':ti,ab,kw OR 'palliative care*':ti,ab,kw OR 'palliative supportive care*':ti,ab,kw OR 'palliative treatment*':ti,ab,kw OR 'palliation':ti,ab,kw OR 'palliative consultation*':ti,ab,kw OR 'palliative medicine*':ti,ab,kw OR 'hospice'/exp OR 'hospice*':ti,ab,kw OR 'palliative nursing'/exp OR 'palliative nursing':ti,ab,kw OR 'terminal care'/de OR 'hospice care'/exp OR 'terminal care*':ti,ab,kw OR 'end-of-life care*':ti,ab,kw OR 'EOL care*':ti,ab,kw) AND (('systematic*':ti,ab,kw AND 'review*':ti,ab,kw) OR ('systematic*':ti,ab,kw AND 'overview*':ti,ab,kw) OR ('systemic*':ti,ab,kw AND 'review*':ti,ab,kw) OR ('systemic*':ti,ab,kw AND 'overview*':ti,ab,kw) OR 'systematic literature review*':ti,ab,kw OR 'Cochrane review*':ti,ab,kw OR 'scoping review*':ti,ab,kw OR 'scoping overview*':ti,ab,kw OR 'scoping literature review*':ti,ab,kw OR 'mapping review*':ti,ab,kw OR 'systematic mapping*':ti,ab,kw OR 'systematical mapping*':ti,ab,kw OR 'umbrella review*':ti,ab,kw OR 'umbrella systematic review*':ti,ab,kw OR 'systematic umbrella review*':ti,ab,kw OR 'umbrella analys*':ti,ab,kw OR 'umbrella of systematic*':ti,ab,kw OR 'review of review*':ti,ab,kw OR 'overview of reviews':ti,ab,kw OR 'overview of systematic reviews':ti,ab,kw OR 'overviews of reviews':ti,ab,kw OR 'reviews in overviews':ti,ab,kw OR 'meta-review*':ti,ab,kw OR 'integrative review*':ti,ab,kw OR 'integrative systematic review*':ti,ab,kw OR 'integrated review*':ti,ab,kw OR 'integrated systematic review*':ti,ab,kw OR 'integrative overview*':ti,ab,kw OR 'meta-synthesis':ti,ab,kw OR 'metasynthesis':ti,ab,kw OR 'research synthesis':ti,ab,kw OR 'meta-ethnography':ti,ab,kw OR 'systematic literature search':ti,ab,kw OR 'systematic literature research':ti,ab,kw OR 'metaanalys*':ti,ab,kw OR 'meta-analys*':ti,ab,kw OR 'meta-analytic review*':ti,ab,kw OR 'meta-analytical review*':ti,ab,kw OR 'meta-analys*':it OR 'systematic review':it) NOT ('letter':it OR 'editorial':it OR 'comment':it OR 'case reports':it OR 'historical article':it OR 'protocol*':ti,ab,kw OR 'withdrawn':ti,ab,kw OR 'retraction of publication':it OR 'publishing'/exp OR 'retracted publication':it OR 'reply':ti,ab,kw OR 'published erratum':it)

**WEB OF SCIENCE (3947 hits, 26/12/2022, 323 met eigen filter)**

**WEB OF SCIENCE (4224 hits, 06/11/2023, beginning - 06/11/2023, 355 hits met eigen filter, update 01/12/2022 - 06/11/2023, 36 hits met eigen filter)**

("schizophreni*" OR "schizoaffective*" OR "schizo-affective*" OR "delusional disorder*" OR "paranoid disorder*" OR "paranoid psychosis" OR "schizotypal disorder*" OR "psychotic disorder*" OR "psychosis" OR "catatoni*" OR "bipolar*" OR "cyclothymi*" OR "depressive disorder*" OR "depressive disease*" OR "depressive illness*" OR "depressive episode*" OR "depressive state*" OR "depressive syndrome*" OR "depression*" OR "unipolar disorder*" OR "manic episode*" OR "mania" OR "affective disorder*" OR "mood disorder*" OR "MDD" OR "disruptive mood dysregulation disorder*" OR "dysthymi*" OR "disthymi*" OR "dysthimi*" OR "premenstrual dysphoric disorder*" OR "severe mental illness*" OR "serious mental illness*" OR "severe mental disorder*" OR "serious mental disorder*") AND ("palliative care*" OR "palliative supportive care*" OR "palliative treatment*" OR "palliative therap*" OR "hospice*" OR "palliative nursing" OR "palliative medicine*" OR "terminal care*" OR "end-of-life care*" OR "EOL care*") AND (("systematic*" AND "review*") OR ("systematic*" AND "overview*") OR ("systemic*" AND "review*") OR ("systemic*" AND "overview*") OR "systematic literature review*" OR "Cochrane review*" OR "scoping review*" OR "scoping overview*" OR "scoping literature review*" OR "mapping review*" OR "systematic mapping*" OR "systematical mapping*" OR "umbrella review*" OR "umbrella systematic review*" OR "systematic umbrella review*" OR "umbrella analys*" OR "umbrella of systematic*" OR "review of review*" OR "overview of reviews" OR "overview of systematic reviews" OR "overviews of reviews" OR "reviews in overviews" OR "meta-review*" OR "integrative review*" OR "integrative systematic review*" OR "integrated review*" OR "integrated systematic review*" OR "integrative overview*" OR "meta-synthesis" OR "metasynthesis" OR "research synthesis" OR "meta-ethnography" OR "systematic literature search" OR "systematic literature research" OR "metaanalys*" OR "meta-analys*" OR "meta-analytic review*" OR "meta-analytical review*" OR "meta-analys*" OR "systematic review") NOT ("letter" OR "editorial" OR "comment" OR "case reports" OR "historical article" OR "protocol*" OR "withdrawn" OR "retraction of publication" OR "retraction of publication as topic" OR "retracted publication" OR "reply" OR "published erratum")

**SCOPUS (3002 hits,** **26/12/2022, 121 met eigen filter)**

**SCOPUS (3226 hits, 06/11/2023, beginning - 06/11/2023, 137 hits met eigen filter, update 01/01/2022 - 06/11/2023, 28 hits met eigen filter)**

(TITLE-ABS("schizophreni*" OR "schizoaffective*" OR "schizo-affective*" OR "delusional disorder*" OR "paranoid disorder*" OR "paranoid psychosis" OR "schizotypal disorder*" OR "psychotic disorder*" OR "psychosis" OR "catatoni*" OR "bipolar*" OR "cyclothymi*" OR "depressive disorder*" OR "depressive disease*" OR "depressive illness*" OR "depressive episode*" OR "depressive state*" OR "depressive syndrome*" OR "depression*" OR "unipolar disorder*" OR "manic episode*" OR "mania" OR "affective disorder*" OR "mood disorder*" OR "MDD" OR "disruptive mood dysregulation disorder*" OR "dysthymi*" OR "disthymi*" OR "dysthimi*" OR "premenstrual dysphoric disorder*" OR "severe mental illness*" OR "serious mental illness*" OR "severe mental disorder*" OR "serious mental disorder*") OR AUTHKEY("schizophreni*" OR "schizoaffective*" OR "schizo-affective*" OR "delusional disorder*" OR "paranoid disorder*" OR "paranoid psychosis" OR "schizotypal disorder*" OR "psychotic disorder*" OR "psychosis" OR "catatoni*" OR "bipolar*" OR "cyclothymi*" OR "depressive disorder*" OR "depressive disease*" OR "depressive illness*" OR "depressive episode*" OR "depressive state*" OR "depressive syndrome*" OR "depression*" OR "unipolar disorder*" OR "manic episode*" OR "mania" OR "affective disorder*" OR "mood disorder*" OR "MDD" OR "disruptive mood dysregulation disorder*" OR "dysthymi*" OR "disthymi*" OR "dysthimi*" OR "premenstrual dysphoric disorder*" OR "severe mental illness*" OR "serious mental illness*" OR "severe mental disorder*" OR "serious mental disorder*")) AND (TITLE-ABS("palliative care*" OR "palliative supportive care*" OR "palliative treatment*" OR "palliative therap*" OR "hospice*" OR "palliative nursing" OR "palliative medicine*" OR "terminal care*" OR "end-of-life care*" OR "EOL care*") OR AUTHKEY("palliative care*" OR "palliative supportive care*" OR "palliative treatment*" OR "palliative therap*" OR "hospice*" OR "palliative nursing" OR "palliative medicine*" OR "terminal care*" OR "end-of-life care*" OR "EOL care*")) AND (TITLE-ABS(("systematic*" AND "review*") OR ("systematic*" AND "overview*") OR ("systemic*" AND "review*") OR ("systemic*" AND "overview*") OR "systematic literature review*" OR "Cochrane review*" OR "scoping review*" OR "scoping overview*" OR "scoping literature review*" OR "mapping review*" OR "systematic mapping*" OR "systematical mapping*" OR "umbrella review*" OR "umbrella systematic review*" OR "systematic umbrella review*" OR "umbrella analys*" OR "umbrella of systematic*" OR "review of review*" OR "overview of reviews" OR "overview of systematic reviews" OR "overviews of reviews" OR "reviews in overviews" OR "meta-review*" OR "integrative review*" OR "integrative systematic review*" OR "integrated review*" OR "integrated systematic review*" OR "integrative overview*" OR "meta-synthesis" OR "metasynthesis" OR "research synthesis" OR "meta-ethnography" OR "systematic literature search" OR "systematic literature research" OR "metaanalys*" OR "meta-analys*" OR "meta-analytic review*" OR "meta-analytical review*" OR "meta-analys*" OR "systematic review") AND NOT ("letter" OR "editorial" OR "comment" OR "case reports" OR "historical article" OR "protocol*" OR "withdrawn" OR "retraction of publication" OR "retraction of publication as topic" OR "retracted publication" OR "reply" OR "published erratum")) OR (AUTHKEY(("systematic*" AND "review*") OR ("systematic*" AND "overview*") OR ("systemic*" AND "review*") OR ("systemic*" AND "overview*") OR "systematic literature review*" OR "Cochrane review*" OR "scoping review*" OR "scoping overview*" OR "scoping literature review*" OR "mapping review*" OR "systematic mapping*" OR "systematical mapping*" OR "umbrella review*" OR "umbrella systematic review*" OR "systematic umbrella review*" OR "umbrella analys*" OR "umbrella of systematic*" OR "review of review*" OR "overview of reviews" OR "overview of systematic reviews" OR "overviews of reviews" OR "reviews in overviews" OR "meta-review*" OR "integrative review*" OR "integrative systematic review*" OR "integrated review*" OR "integrated systematic review*" OR "integrative overview*" OR "meta-synthesis" OR "metasynthesis" OR "research synthesis" OR "meta-ethnography" OR "systematic literature search" OR "systematic literature research" OR "metaanalys*" OR "meta-analys*" OR "meta-analytic review*" OR "meta-analytical review*" OR "meta-analys*" OR "systematic review") AND NOT ("letter" OR "editorial" OR "comment" OR "case reports" OR "historical article" OR "protocol*" OR "withdrawn" OR "retraction of publication" OR "retraction of publication as topic" OR "retracted publication" OR "reply" OR "published erratum"))

**CINAHL (3613 hits zonder filter, 88 hits met eigen filter, 26/12/2022)**

**CINAHL (3668 hits, 06/11/2023, beginning - 06/11/2023, 92 hits met eigen filter, update 01/01/2022 - 06/11/2023, 12 hits met eigen filter)**

((MH "Psychotic Disorders+") OR (MH "Affective Disorders+") OR TI("schizophreni*" OR "schizoaffective*" OR "schizo-affective*" OR "delusional disorder*" OR "paranoid disorder*" OR "schizotypal disorder*" OR "psychotic disorder*" OR "psychosis" OR "catatoni*" OR "bipolar and related disorder*" OR "bipolar disorder*" OR "bipolar I disorder*" OR "bipolar type I disorder*" OR "bipolar II disorder*" OR "bipolar type II disorder*" OR "cyclothymi*" OR "manic depressi*" OR "manic episode*" OR "mania" OR "affective disorder*" OR "bipolar illness" OR "mood disorder*" OR "depressive disorder*" OR "major depression" OR "MDD" OR "agitated depression" OR "reactive depression" OR "vital depression" OR "disruptive mood dysregulation disorder*" OR "dysthymi*" OR "disthymi*" OR "dysthimi*" OR "premenstrual dysphoric disorder*" OR "severe mental illness*" OR "serious mental illness*" OR "severe mental disorder*" OR "serious mental disorder*")) AND ((MH "Palliative Care") OR (MH "Hospices") OR (MM "Hospice Patients") OR (MM "Hospice and Palliative Nursing") OR (MM "Hospice Care") OR (MM "Palliative Medicine") OR (MH "Terminal Care") OR TI("palliative care*" OR "palliative supportive care*" OR "palliative treatment*" OR "palliative therap*" OR "hospice*" OR "palliative nursing" OR "palliative medicine*" OR "terminal care*" OR "end-of-life care*" OR "EOL care*")) AND ((MM "Meta Analysis") OR (MM "Systematic Review") OR TI(("systematic*" AND "review*") OR ("systematic*" AND "overview*") OR ("systemic*" AND "review*") OR ("systemic*" AND "overview*") OR "systematic literature review*" OR "Cochrane review*" OR "scoping review*" OR "scoping overview*" OR "scoping literature review*" OR "mapping review*" OR "systematic mapping*" OR "systematical mapping*" OR "umbrella review*" OR "umbrella systematic review*" OR "systematic umbrella review*" OR "umbrella analys*" OR "umbrella of systematic*" OR "review of review*" OR "overview of reviews" OR "overview of systematic reviews" OR "overviews of reviews" OR "reviews in overviews" OR "meta-review*" OR "integrative review*" OR "integrative systematic review*" OR "integrated review*" OR "integrated systematic review*" OR "integrative overview*" OR "meta-synthesis" OR "metasynthesis" OR "research synthesis" OR "meta-ethnography" OR "systematic literature search" OR "systematic literature research" OR "metaanalys*" OR "meta-analys*" OR "meta-analytic review*" OR "meta-analytical review*" OR "meta-analys*" OR "systematic review") NOT ("letter" OR "editorial" OR "comment" OR "case reports" OR "historical article" OR "protocol*" OR "withdrawn" OR "retraction of publication" OR "retraction of publication as topic" OR "retracted publication" OR "reply" OR "published erratum"))
