# Supplementary material for: End-of-life care for people with severe and persistent mental illness and a life-limiting disease: An umbrella review
Source: Eur Psychiatry. 2025 Mar 24;68(1):e49. doi: 10.1192/j.eurpsy.2025.2440 (PMC12041735; doi:10.1192/j.eurpsy.2025.2440)
Supplement: Denduyver et al. supplementary material 2 — Denduyver et al. supplementary material [file S092493382502440Xsup002.docx]

|  | Boschen et al. | Hannigan et al. | Riley et al. | Baruth et al. | Hanan et al. | Wilson et al. | Den Boer et al. | Donald et al. | Relyea et al. | Woods et al. |
| --- | --- | --- | --- | --- | --- | --- | --- | --- | --- | --- |
| Review question explicitly stated | YES | YES | YES | YES | YES | YES | YES | YES | YES | YES |
| Appropriate inclusion criteria | YES | YES | YES | YES | YES | YES | YES | YES | YES | YES |
| Appropriate search strategy | YES | YES | YES | YES | NO | YES | YES | YES | YES | YES |
| Adequate sources | YES | YES | YES | YES | YES | YES | YES | YES | YES | YES |
| Appropriate criteria for appraisal | NO | YES | YES | NO | NO | YES | YES | NO | NO | UNCLEAR |
| Independently conducted appraisal | NO | YES | YES | NO | NO | YES | YES | NO | NO | UNCLEAR |
| Methods to minimize errors in data extraction | YES | YES | NO | NO | NO | YES | YES | YES | YES | UNCLEAR |
| Appropriate methods to combine studies | YES | YES | YES | YES | YES | YES | YES | YES | YES | YES |
| Assessment of likelihood of publication bias | NOT APPLICABLE | NOT APPL. | NOT APPL. | NOT APPL. | NOT APPL. | NOT APPL. | NOT APPL. | NOT APPL. | NOT APPL. | NOT APPL. |
| Recommendations supported by reported data | NO | YES | NO | YES | YES | NO | YES | YES | YES | YES |
| Appropriate directives for new research | YES | YES | YES | NO | YES | YES | YES | YES | YES | YES |
